# Diphtheria Not a Sewer-Gas Disease

**Published:** 1886-09

**Authors:** 


### *DIPHTHERIA NOT A SEWER-GAS DISEASE.*

Under this title, the *Medical Record*, May 1, 1886 (Editorial), quotes from Dr. Erwin F. Smith's paper (Report Michigan State Board of Health, 1885), in which he claims to establish the following propositions: "(1) Diphtheria is as frequent in the country as in the city; i. e., in non-sewered as in sewered districts. (2) Diphtheria has been more frequent and fatal in certain rural districts than in any city whatsoever. (3) Diphtheria is not more frequent or fatal in sewered cities than in unsewered ones. (4) Of two given cities, equally well or ill-sewered, diphtheria, during a long series of years, may be widely prevalent in the one and rare in the other. (5) Certain sewered cities have never suffered seriously from diphtheria, while others have been afflicted very much worse in recent years (i. e., since the houses have been protected from sewer-air) than formerly, when with the same sewers, but much less perfect plumbing, flushing and ventilation, the sewer-air found its way into a majority of the houses. (6) When an epidemic of diphtheria appears in a city, the sewered and unsewered portion generally suffer alike. (7) No relation of interdependence can be traced between diphtheria and the sanitary state of a city, such, for example, as enables us to predict with almost absolute certainty, the typhoid fever mortality of a city from a knowledge of its sanitary condition, or conversely, the sanitary condition from its typhoid mortality. (8) The annual mortality from diphtheria fluctuates greatly, and this, too, in cities where the sanitary conditions are very nearly constant. (9) Diphtheria is a disease of cold weather, being most active when putrefactive decomposition in sewers is presumably least so. (10) Diphtheria is a contagious disease, transmissible from person to person and place to place, like small-pox and scarlet fever. (11) The closing of schools and other places of public gathering checks an epidemic; and the isolation of the sick from the well, with the subsequent proper disinfection of the sick-room and its contents, extinguishes it. (12) The data to be relied upon to prove a connection between sewerage and diphtheria either cover too short a period to be trustworthy, or are drawn from single cities having incomplete and defective sewerage.

"If these propositions be true, it follows as a necessary corollary that there is no direct relation between sewers and diphtheria." These conclusions are basted upon a study of the vital statistics of a large number of European and American cities and districts.—*Fort Wayne Medical Journal.*

